# Adaptive tolerance to a pathogenic fungus drives major histocompatibility complex evolution in natural amphibian populations

**DOI:** 10.1098/rspb.2015.3115

**Published:** 2016-03-30

**Authors:** Anna E. Savage, Kelly R. Zamudio

**Affiliations:** Department of Ecology and Evolutionary Biology, Cornell University, Corson Hall, Ithaca, NY 14853, USA

**Keywords:** major histocompatibility complex, amphibian, chytridiomycosis, *Batrachochytrium dendrobatidis*, immunogenetics, adaptation

## Abstract

Amphibians have been affected globally by the disease chytridiomycosis, caused by the fungus *Batrachochytrium dendrobatidis* (*Bd*), and we are just now beginning to understand how immunogenetic variability contributes to disease susceptibility. Lineages of an expressed major histocompatibility complex (MHC) class II locus involved in acquired immunity are associated with chytridiomycosis susceptibility in controlled laboratory challenge assays. Here, we extend these findings to natural populations that vary both in exposure and response to *Bd*. We find that MHC alleles and supertypes associated with *Bd* survival in the field show a molecular signal of positive selection, while those associated with susceptibility do not, supporting the hypothesis that heritable *Bd* tolerance is rapidly evolving. We compare MHC supertypes to neutral loci to demonstrate where selection versus demography is shaping MHC variability. One population with *Bd* tolerance in nature shows a significant signal of directional selection for the same allele (allele Q) that was significantly associated with survival in an earlier laboratory study. Our findings indicate that selective pressure for *Bd* survival drives rapid immunogenetic adaptation in some natural populations, despite differences in environment and demography. Our field-based analysis of immunogenetic variation confirms that natural amphibian populations have the evolutionary potential to adapt to chytridiomycosis.

## 1. Introduction

Emerging infectious diseases are key threats to wildlife populations [1] that often have complex, varied, or uncertain causes [2]. Fungal diseases in particular are on the rise [3], thus understanding the mechanisms of immune system response to fungal pathogens is of particular importance for predicting whether wildlife populations can adapt to novel infections. The amphibian disease chytridiomycosis, caused by the fungus *Batrachochytrium dendrobatidis* (*Bd*), has resulted in the decline or extinction of hundreds of species worldwide [3–5]. Amphibian species demonstrate a wide range of responses to chytridiomycosis [6] that are largely driven by environment, ecology, and life history [7–10]. Controlled laboratory experiments show that host immunological responses also contribute to *Bd* resistance [11–15], but it has proved difficult to quantify differences in susceptibility among species or natural populations because of the confounding effects of environment, pathogen dynamics, and host demographic factors contributing to disease [16–18]. Thus, the potential evolution of host resistance in response to this emergent disease remains largely unexplored in natural populations.

Amphibian immune systems are structurally and functionally similar to other vertebrates in possessing innate and acquired immune pathways [19]. One important host immune component contributing to *Bd* responses is the major histocompatibility complex (MHC), a family of immune-related genes conserved across vertebrates [20]. Class I and II MHC molecules bind pathogen molecules on their peptide-binding regions (PBRs) and present them to T-cells to initiate an acquired immune response [21]. This central role in initiating immunity creates strong selection on MHC loci for numerous polymorphisms and gene copies, thereby maximizing the array of pathogens that can be recognized [22,23]. Class II MHC genes are expressed on immune surveillance cells in amphibian skin [19,24] and typically recognize bacterial and fungal pathogens, whereas class I molecules are involved primarily in viral immunity and self-discrimination [25]. Class II loci are, therefore, ideal targets for the study of immunogenetic responses to chytridiomycosis, a fungal disease that infects amphibian epidermal cells [3].

Natural wildlife populations show correlations between MHC polymorphism and disease susceptibility [22]. Four non-exclusive evolutionary mechanisms potentially explain MHC allele distributions after pathogen-imposed selection in populations. First, overdominance may arise if MHC heterozygotes are able to bind a wider inventory of antigens [26], resulting in higher fitness compared with homozygotes [27]. Second, directional selection may occur if a specific allelic lineage that confers resistance to a common pathogen increases in frequency over successive generations [28,29]. Third, frequency-dependent selection may occur when pathogens become adapted to the most common host genotype and rare MHC alleles confer a selective advantage until they become common [30–32]. Finally, diversifying selection for numerous resistance-conferring alleles within a spatially heterogeneous selective landscape [33] may cause balanced MHC polymorphism, a pattern that is indistinguishable from frequency-dependent selection [22,34]. Each of these mechanisms have probably shaped MHC diversity over the history of natural populations; thus, teasing apart the specific immunogenetic consequences of *Bd*-imposed selection will require multiple lines of evidence and the ability to distinguish historical versus recent selective events.

In anurans, the MHC genomic region has been characterized in two model species, *Xenopus laevis* [35,36] and *Silurana tropicalis* [37]. Both species have the ancestral tetrapod MHC gene organization [38,39] and diverged early in the anuran phylogeny [40]. Experimental studies in *Silurana* find that under some conditions, *Bd* infection activates innate immune defences [41] or minimal immune responses [42], while under other conditions, acquired immunity is induced [14]. Interestingly, the *Bd*-susceptible species *Rana muscosa* and *R. sierrae*, are similar to *Silurana* in that they show no evidence of a robust immune response [42]. By contrast, the highly susceptible *Atelopus zeteki* mounts both innate and acquired immune defences against *Bd* in challenge experiments, but these efforts are not protective and previous *Bd* exposure does not increase survival [43]. In other species, exposure to *Bd* increases subsequent immunity; previous *Bd* exposure in *Osteopilus septentrionalis* decreased pathogen burden and increased lymphocyte proliferation and survival [14]. *Bd* also potentially suppresses effective acquired immune responses. Anuran T- and B-cells are killed by *Bd in vitro* [13], and expression of T-cell pathway genes are suppressed in experimentally *Bd* infected individuals compared with controls in four frog species [44]. Uncertainty thus remains over the necessary immune system components, antigenic targets, and particularly the gene by environment interactions that lead to an effective immune response against *Bd*.

Variation in MHC genes has been characterized in natural amphibian populations that differ in susceptibility to non-fungal pathogens [28,45,46] as well as *Bd* [12,15]. In *Bufo calamita*, class II genotype frequencies varied in a pattern consistent with directional selection in response to pathogen prevalence among populations [12], and a comparison of class II diversity across nine amphibian genera with *Bd* susceptibility data found that more resistant species and populations have common amino acids in peptide-binding pockets [15]. Combined, these studies indicate a functional role for MHC genes in natural chytridiomycosis dynamics.

*Lithobates yavapaiensis* is a North American frog that has declined due to seasonal chytridiomycosis outbreaks since at least 1990 [12,47]. Our earlier experimental *Bd* infections of laboratory-reared *L. yavapaiensis* from five natural populations identified specific class II MHC genotypes that were associated with survival within and among populations [11]. Both MHC heterozygotes and individuals bearing MHC allele Q had significantly higher probabilities of surviving *Bd* infection [48]. Bataille *et al.* [15] subsequently extended these findings with experimental *Bd* infections of the Australian tree frog *Litoria verreauxii alpina*, and survival was significantly associated with MHC alleles with amino acid substitutions in the same region where we detected positive selection acting on allele Q. Here, we test the generality of our experimental infection results in natural populations, and ask whether the same signatures of selection and immunogenetic predictors of susceptibility can be found among individuals from populations that differ in their demographic responses to *Bd*. We characterize class II MHC in field-sampled frogs from eight populations currently infected with *Bd*, and interpret genetic variation at this locus in the light of our published multi-year seasonal field estimates of population and individual *Bd* susceptibilities [11,16]. We also compare neutral genetic markers with immunogenetic genotypes to identify significant signals of natural selection in response to chytridiomycosis. We extend the experimental finding that immunogenetic variation determines *Bd* susceptibility by elucidating the mechanisms of evolutionary response to disease across a variable ecological and environmental landscape, predicting the potential for evolution of resistance in natural populations.

## 2. Material and methods

### (a) Field surveys

We surveyed eight *L. yavapaiensis* populations for *Bd* and chytridiomycosis during winter months (January–February) of 2007–2011 [11,16], the time of year when *Bd* mortalities occur in this species [16]. Using standardized protocols [49], we swabbed the epidermis of all individuals and used quantitative PCR to measure infection intensity (the number of *Bd* genome equivalents (GE) recovered per swab). We categorized each dead individual that tested positive for *Bd* infection as a chytridiomycosis mortality event. Additionally, we collected any individual with signs of chytridiomycosis (skin redness, lethargy, failure to seek shelter, and loss of righting ability) for overnight observation, and also categorized these as chytridiomycosis-induced mortality events if death occurred within 24 h and the individual tested positive for *Bd*. All eight populations were surveyed greater than or equal to two times per winter, and because *L. yavapaiensis* is a stream-dwelling species, inhabiting shallow flowing water and small plunge pools, the sites could be exhaustively screened for dead and dying individuals. Populations could, therefore, be definitively characterized for *Bd* susceptibility based on the number of frogs found dead or dying with *Bd* infection. By contrast, the ultimate fate of individuals sampled alive could not be determined for the five populations with mortality (i.e. they could hypothetically develop chytrydiomycosis at a later time point). Our analyses thus measure genetic correlates of *Bd*-susceptible frogs rather than directly assessing *Bd* survival. We estimated *Bd* and chytridiomycosis mortality prevalence and 95% Clopper–Pearson binomial confidence intervals [50] for each *L. yavapaiensis* population.

### (b) Microsatellite genotyping

We previously genotyped 128 *L. yavapaiensis* individuals at 14 unlinked microsatellite loci using published protocols [16,17,51]. We used GENEPOP v. 3.4 [48] to calculate observed and expected heterozygosity and test for deviations from Hardy–Weinberg equilibrium at each locus and population locality using a Monte-Carlo chain method (1 000 dememorizations, 100 batches, 1 000 iterations) [52] with Bonferroni correction for multiple tests (adjusted *p* = 0.00022).

### (c) Major histocompatibility complex amplification, cloning, and sequencing

We extracted genomic DNA from ethanol-preserved toe clips from the same 128 individuals that were microsatellite genotyped. The majority (108) of individuals were sampled in winter, but for populations with prohibitively small winter sample sizes (CIC, TV, and WC), we sampled 20 additional frogs collected in the summers of 2006–2007. We used a degenerate forward primer [45] and a ranid frog intron-specific reverse primer [11,46] to amplify 249 base pairs (bp) of exon 2 (which encompasses the peptide-binding region) and 189 bp of adjacent 3′-flanking intron of an expressed MHC class II locus. We used previously published protocols to amplify, clone, and sequence MHC alleles [11,46]. We screened each MHC sequence and only retained those obtained from at least two clones. For each individual, sequences recovered only once with less than or equal to two nucleotide differences to other cloned sequences were attributed to PCR/cloning errors and discarded. After excluding these sequences, no more than two unique MHC alleles were recovered from any individual. To assess the frequency of artefactual alleles arising from cloning, we also used the same MHC primers with 454 adapters added to the 5′ ends (fusion primers) to perform amplicon sequencing on a GS Junior (Roche) using two different PCR amplifications and more than 40× depth of sequencing for a subset of our sampled individuals (*N* = 29 frogs from five populations). We multiplexed individuals by using a suite of 8 bp unique sequence tags added to the 454 fusion primers that differed from each other in at least three positions to minimize misassignment. PCR amplifications were performed in 20 µl, including 3 µl of each primer, 3 µl of DNA, and 7.5 µl of HotStar PCR Master Mix (Qiagen). We pooled equimolar quantities of PCR products amplified with distinct fusion primers, purified DNA with a MinElute PCR Purification Kit (Qiagen) and sequenced the pool of amplicon samples from all individuals on a GS Junior run (Roche). All individuals were amplified and sequenced at least twice to ensure we did not generate any artificial alleles resulting from PCR or sequencing error. We used Newbler v. 2.6 (Roche) to remove adapter sequences and assemble MHC amplicons into contigs. All raw MHC amplicon reads were analysed with jMHC v. 1.0 to demultiplex samples and assign genotypes. Only reads that had two complete barcodes and a perfect match to the primer barcode were retained and assigned to their respective sample and repetition number, and only alleles that were found in at least two separate samples or two separate repetitions with a minimum of 15 reads were considered real. We aligned all MHC alleles using Sequencher (Gene Codes Corporation) with adjustment by eye and compared cloning-derived with 454-derived alleles across individuals.

### (d) Genealogy reconstruction

We tested the MHC alignment for evidence of recombination using the single breakpoint method [53] before performing a Bayesian analysis to reconstruct genealogical relationships among alleles. We used class II exon 2 sequences from *X. laevis* and *S. tropicalis* (GenBank accession numbers NM_001114771 and NM_001045794) as outgroups. Model parameters were determined using the Akaike information criteria in jModeltest [54]. We used the best-fit model (general time reversible (GTR) model with invariable sites plus gamma (I + γ) distribution (GTR + I + G)) to estimate a 95% credible set of rooted MHC genealogies in the software MrBayes 3.1 [55]. We ran two separate analyses in MrBayes for 1 × 10^7^ generations and sampled every 500th generation of the Markov chain. We used Tracer v. 1.4 to assess stationarity of model parameters, convergence of model parameters between runs, the number of burn-in samples, and the effective sample sizes for each parameter.

### (e) Tests of selection

We ran tests of selection using HyPhy [56] with the Bayesian genealogy as our input tree, excluding outgroup sequences. We used PARRIS to test for positive selection in the entire alignment [57], evolutionary fingerprinting to infer the number of positive selection rate classes and the intensity of selection in each rate class [58], and the most conservative maximum-likelihood method (SLAC) to test for residue-specific positive selection across lineages [59].

### (f) Major histocompatibility complex supertyping

To collapse MHC alleles into functional supertypes, we extracted the 13 codon positions in our MHC alignment known to affect peptide-binding capabilities of human class II alleles [15,21] and then characterized each site based on five physio-chemical descriptor variables: z1 (hydrophobicity), z2 (steric bulk), z3 (polarity), z4 and z5 (electronic effects) [60]. We used discriminant analysis of principle components to define functional genetic clusters using the adegenet 1.4-0 package in R [61], which implements a *k*-means clustering algorithm using the Bayesian information criterion (BIC). The optimal number of clusters was determined using ΔBIC ≤ 2, and alleles within clusters were collapsed into a single MHC supertype.

### (g) Selection and genetic differentiation among populations

We used software for the measurement of genetic diversity (SMOGD) [62] to estimate *D* [63] across all population pairs for (i) 14-locus microsatellite genotypes, (ii) MHC exon 2 genotypes, and (iii) MHC supertypes. We also calculated observed and expected heterozygosity, nucleotide diversity (*π*), and theta (*θ*), and performed Ewens–Watterson (E-W) tests on MHC exon and intron genotypes using Arlequin v. 3.5 [64]. We used the lm function in R [65] to perform linear regression on all pairwise population measures of *D* from MHC supertype versus microsatellite genotypes. Significant outliers were identified as data points with both Cook's *D* > 4/*n* and leverage values > 3/*n*, where *n* is the number of observations [66].

### (h) Statistical analyses

Differences in *Bd* infection intensity and nucleotide diversity across populations were assessed using Student's *t*-tests implemented in JMP software, v. 9.0 (SAS). Differences in supertype frequencies across individuals that were alive versus dead at the time of sampling were inferred using 95% Clopper–Pearson binomial confidence intervals. We used a previously published protocol [11] to calculate the relative risk (RR) for MHC supertypes and for the three alleles recovered in both the field and laboratory study.

## 3. Results

### (a) *Batrachochytrium dendrobatidis* infection dynamics vary within and among populations

**Figure 1.**
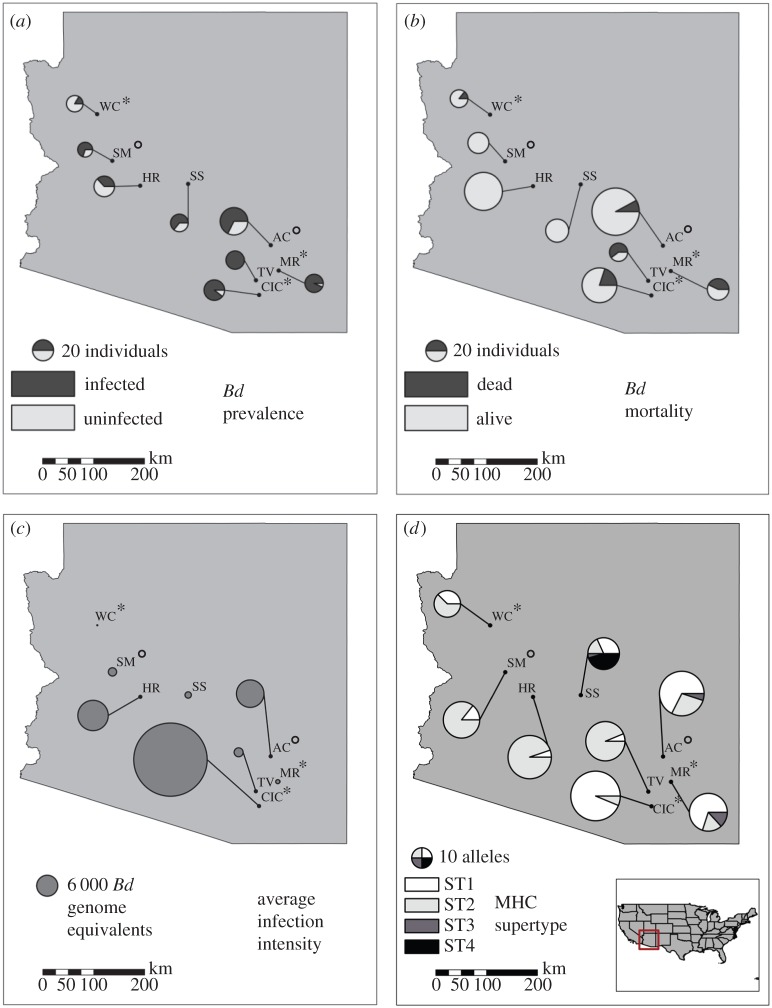
*Batrachochytrium dendrobatidis* (*Bd*) infection dynamics and immunogenetics among eight *Lithobates yavapaiensis* populations sampled in winter. Asterisks represent populations with 100% mortality and circles represent populations with less than 100% mortality in previous controlled laboratory infections [11]. (*a*) Proportion of individuals infected with *Bd*, (*b*) proportion of individuals found dead versus alive, (*c*) average *Bd* infection intensity among *Bd-*positive frogs, and (*d*) MHC supertype frequencies. Circle sizes are proportional to sample size, mean infection intensity, and allele frequency, respectively. AC, Aravaipa Canyon; CIC, Cienega Creek; HR, Hassayampa River; MR, Muleshoe Ranch; SM, Santa Maria River; SS, Seven Springs; TV, Tanque Verde Canyon; WC, Willow Creek. (Online version in colour.)

*Lithobates yavapaiensis* individuals varied within and among the eight field-sampled populations in *Bd* infection prevalence (figure 1*a*), associated mortality (figure 1*b*), and infection intensity (figure 1*c*) [16]. Three populations were *Bd* tolerant, with no winter mortality (HR, SM, and SS) despite high winter *Bd* infection prevalence and intensity. For the five populations with both laboratory and field data, mortality was consistently higher in our experimental infection study (41–100% mortality [11]) than in the field (figure 1*b*). Within populations, frogs that died had significantly higher infection intensities than frogs that survived (two-tailed paired Student's *t*-test, *p* = 0.046).

### (b) Major histocompatibility complex variation is associated with *Batrachochytrium dendrobatidis* susceptibility in the wild

**Figure 2.**
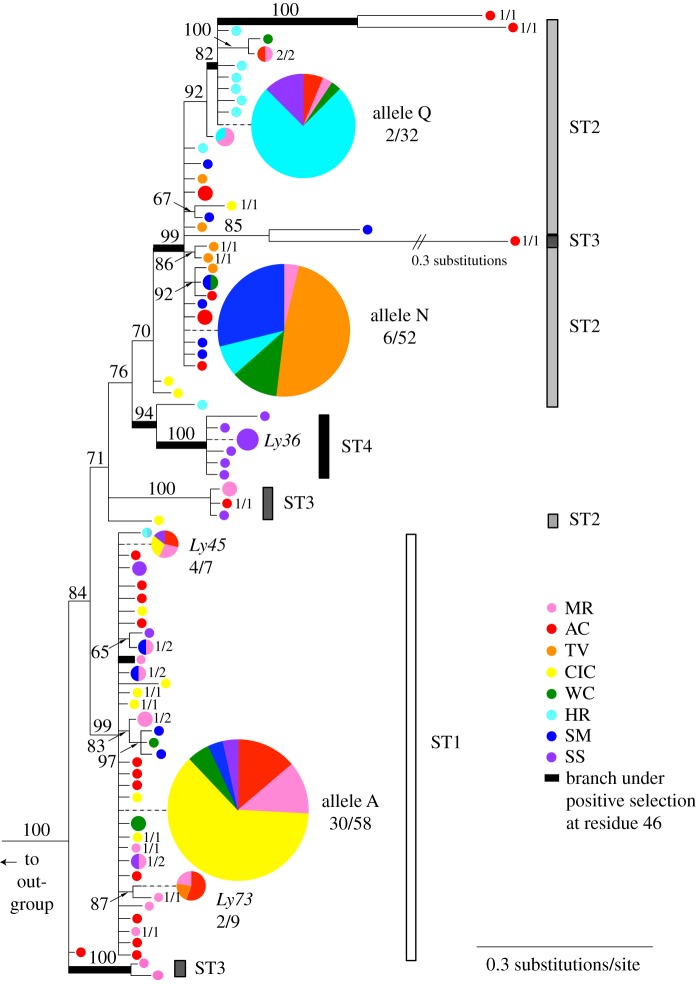
Maximum-likelihood genealogy of 84 recovered MHC alleles from eight *L. yavapaiensis* populations comprising four functional MHC supertypes (ST1–ST4). Branches with significantly elevated non-synonymous substitution rates for codon 46 are shown in bold. Posterior probabilities (PP) > 70% are shown, population name abbreviations follow figure 1, and circle size is proportional to allele frequency. Dashed lines indicate spacing for presentation purposes and are not branches.

We identified 84 unique MHC class II PBR alleles among the eight sampled populations (figure 2). Three PBR alleles were recovered at high frequency (alleles A, N, and Q) and are the same three alleles that were common in our experimental infection study [11]. Sixty-six of the 84 alleles were recovered from a single individual. Owing to the high proportion of singletons and knowledge that a subset of codon sites within exon 2 are responsible for peptide-binding capabilities of the MHC molecule [15,21], we converted alleles into four MHC supertypes based on physio-chemical binding properties (figure 1*d*). For the subset of individuals genotyped using both cloning and 454 amplicon sequencing, MHC alleles from 26/29 individuals (90%) were identical between methods. Of the individuals that were not identical, one individual was mismatched for one allele only, and two individuals were mismatched for both alleles (electronic supplementary material, S1). After converting alleles into four functional supertypes (electronic supplementary material, S2), none of these five mismatched alleles resulted in differences in MHC supertype assignments. Therefore, we conclude that only a small proportion of cloned alleles in this study are artefactual, and that any undetected error derived from cloning is minimal once analysed as MHC supertypes.

**Figure 3.**
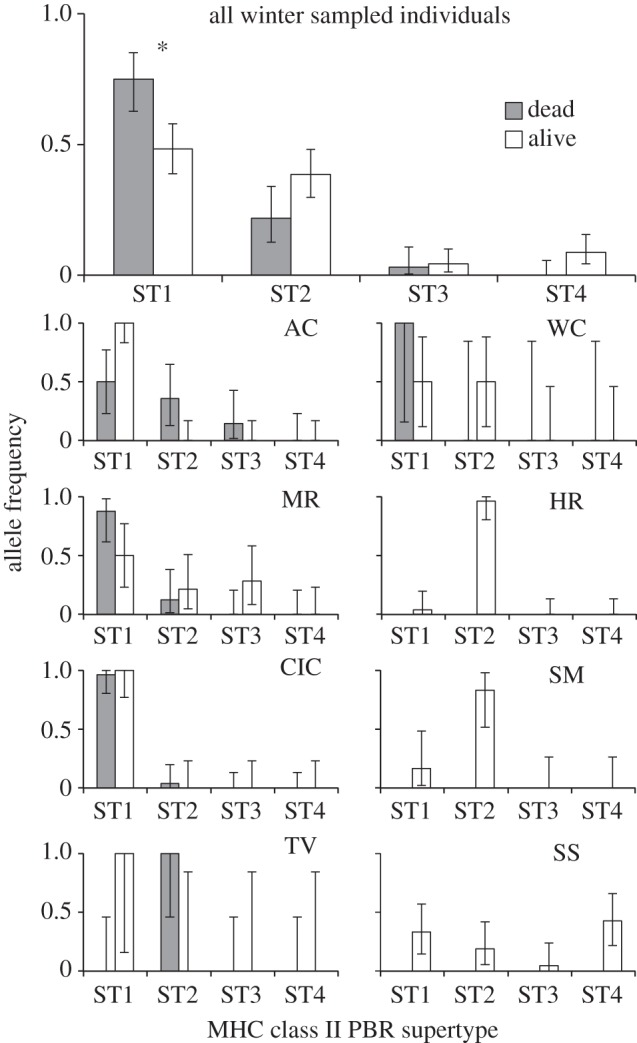
MHC supertype allele distributions for individuals sampled alive (white bars) versus dead (grey bars) across all individuals sampled in winter (upper panel) and within each population (lower panels). Error bars represent 95% binomial confidence intervals for proportions. Significantly different supertype frequencies between alive versus dead frogs are show with asterisks. Population name abbreviations follow figure 1.

The MHC supertypes ST1 and ST4 show a phylogenetic signal, with ST1 comprising all alleles in the clade including allele A, and ST4 comprising all alleles in a clade unique to population SS (figure 2). By contrast, ST2 includes most of the clade with alleles N and Q, but ST3 and ST4 render it paraphyletic, and ST3 is distributed broadly throughout the genealogy. Only MHC ST1 was significantly associated with susceptibility (figure 3; electronic supplementary material, S3); individuals with ST1 had nearly a threefold increased risk of death (RR = 2.8, *p* = 0.004). Notably, allele A (the dominant allele within ST1) was also significantly associated with mortality both in our field-collected samples (RR = 3.2, *p* < 0.0001) and in our experimental infection study [11]. ST4, which was only present in six frogs sampled alive from population SS, showed a trend towards a reduced risk of mortality (RR = 0, *p* = 0.06). ST2 and ST3 were not significantly associated with susceptibility or survival, nor were ST heterozygotes, despite a significant heterozygote survival advantage in our experimental infection study [11]. Allele Q was significantly associated with survival both in our field-collected samples (RR = 0.23, *p* = 0.008) and in our earlier experimental infection study [11].

### (c) Positive selection drives major histocompatibility complex evolution at a peptide-binding codon

Evolutionary models that included positive selection (a ratio of non-synonymous nucleotide substitutions per non-synonymous sites (dN) to synonymous substitutions per synonymous sites (dS), or *ω*, > 1) fit the alignment of PBR sequences significantly better than models excluding positive selection (evolutionary fingerprinting, log likelihood = −1951.54, *p* < 0.001). The best-fit model included three nucleotide substitution rate classes, and found that 65% of codons experienced negative selection (*ω* < 1), 30% experienced moderate positive selection (*ω* = 1.28), and 5% experienced strong positive selection (*ω* = 1.95). In codon-specific tests of selection, we detected positive selection acting on one codon: position 46 of the MHC alignment (*p* = 0.05, normalized dN–dS = 4.08; bold branches in figure 2), which is among the 13 codon positions that determine human MHC peptide-binding [21]. Codon 46 is also the only residue where a significant signal of positive selection was detected in our experimental infection study [11].

### (d) Selection and not demography drives major histocompatibility complex population differentiation

**Figure 4.**
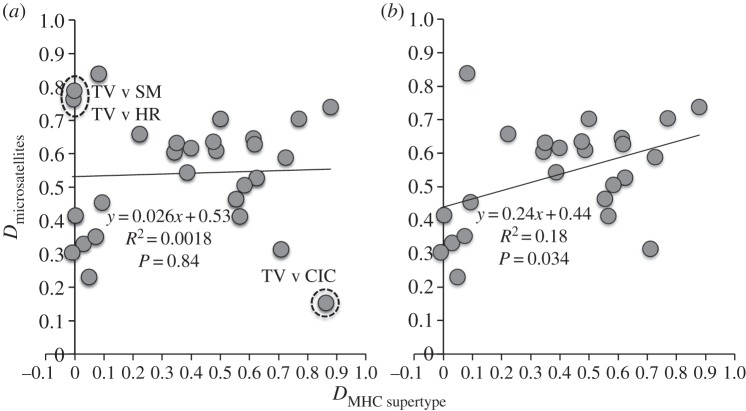
Population differentiation (*D*) inferred from MHC supertypes versus 14 microsatellite markers demonstrates that both selective and demographic processes shape MHC evolution. Regression of MHC and microsatellite pairwise population *D* values for (*a*) all pairwise comparisons and (*b*) after removing three significant outliers. Outliers are encompassed within dashed circles in *a*, and the comparisons they represent are shown using population abbreviations from figure 1.

To disentangle signatures of selection from demography, we compared MHC supertype frequencies with 14 putatively neutral microsatellite loci (figure 4). The average population genetic differentiation (*D*) was significantly higher when measured from microsatellites (*D* = 0.54) compared with MHC supertypes (*D* = 0.39; two-tailed paired Student's *t*-test, *p* = 0.025). Population pairwise estimates of *D* inferred from microsatellites were not significantly correlated to those from MHC supertypes (figure 4*a*), indicating discordance between MHC and neutral genetic differentiation. Three comparisons between TV and other populations were identified as significant regression outliers (figure 3*a*, dashed circles), and removing these three data points produced a significant positive correlation between *D* measured from microsatellites and MHC supertypes (figure 4*b*).

Genetic diversity was significantly higher for MHC exons compared with introns when measured either as nucleotide diversity (*π*; two-tailed paired Student's *t*-test, *p* = 0.014) or the number of segregating sites (*θ*; two-tailed paired Student's *t*-test, *p* = 0.0097; electronic supplementary material, S4). We detected significant signatures of directional selection from MHC heterozygosity but not from microsatellite heterozygosity in populations CIC (20% mortality) and TV (60% mortality), indicating directional selection acts on MHC in these two populations. By contrast, AC (8% mortality), HR (0% mortality), and SM (0% mortality) had significant signatures of directional selection from both MHC and microsatellite heterozygosities, indicating recent demographic expansion rather than selection may shape contemporary MHC evolution in those populations. Neither locus type produced a significant signature of directional or balancing selection in populations SS, WC, or MR (electronic supplementary material, S4).

## 4. Discussion

Whether natural populations can rapidly adapt to novel pathogens remains a critically important question for evolutionary biology in an era of emerging infectious diseases. *L. yavapaiensis* has declined in recent decades due to chytridiomycosis outbreaks, habitat loss, and invasive species [47,67]. The seasonal selective pressure imposed by *Bd* each winter [16,17] means that populations unable to adapt will probably become extirpated. Here, we find that some populations may be adapting to *Bd* via standing MHC variation [68] that confers survival (namely, allele Q and ST4), while other populations lacking these MHC variants may not have the immunogenetic variation necessary to adapt. Positive selection was only detected along lineages leading to survival-associated or neutral alleles and supertypes (figure 2), but not the susceptibility-associated ST1. Thus, at the time of initial chytridiomycosis outbreaks, populations with standing MHC variation that included survival alleles have evolved partial (AC, 8% mortality) or complete (HR, SM, SS, no mortality) *Bd* tolerance. By contrast, populations with high frequencies of the susceptibility supertype ST1 (CIC, MR, TV, WC) were probably decimated by initial chytridiomycosis outbreaks and now have small population sizes, limited genetic diversity, and struggle to persist in the face of repeated bouts of *Bd* mortality [16,17]. MR is an exception, maintaining high MHC diversity (including allele Q) and high mortality (figure 3). However, this pattern is consistent with previous analyses of this population, which is adjacent to a thermal spring. Here, warm water protects the source population from extirpation but also prevents selection from acting to increase the frequency of survival alleles, creating an ongoing source-sink dynamic when juveniles migrate out from the thermal spring pools and develop chytridiomycosis [16,17]. Finally, in addition to selection acting on standing genetic variants, novel MHC variants providing a *Bd* survival advantage may have arisen in one population. SS had no observed *Bd* mortality and a low frequency of allele Q, but is the only population with individuals bearing ST4, a recently diversified clade with a significant signature of positive selection (figure 2). This pattern suggests that adaptation to *Bd* may have evolved rapidly in population SS from *de novo* genetic mutations, a less frequently documented phenomenon [69].

By using field data to validate a controlled experimental study, our analyses collectively show that selection caused by chytridiomycosis has contributed to the evolution of an expressed class II MHC locus in *L. yavapaiensis*. Our sampling from wild populations confirms three of the immunogenetic disease association patterns that were previously recovered in our experimental infection study [11]: individuals with ST1/allele A had a significantly increased risk of *Bd* mortality, those with allele Q had a significantly reduced risk of mortality (figure 3), and we found a significant signature of selection acting on the same peptide-interacting codon in both studies (figure 2). By contrast, here we found no evidence of heterozygote advantage when examining MHC genotypes in natural population samples, despite a strong survival advantage of heterozygotes in our experimental infection study [11]. Because we could only definitively assess mortality events, whereas *Bd* survivors may have occasionally been mis-identified among individuals developing chytridiomycosis exceptionally late in the season after surveys took place, MHC associations to susceptibility are stronger than associations to tolerance. Thus, an MHC-based heterozygote advantage may exist and could be uncovered with finer-scale field sampling or additional experimental infection studies. Alternately, because the previous laboratory study used full- and half-sibling clutches to represent populations, the apparent signal of overdominance may have been driven by the allelic composition of heterozygous individuals; all survivors with allele Q were heterozygotes. Indeed, experimental infection studies examining resistance to single pathogens rather than general pathogen-fighting ability find evidence for survival based on particular MHC alleles rather than heterozygotes [70].

Both directional [71] and diversifying [72] selection on MHC alleles are commonly observed evolutionary responses to pathogen pressure in natural populations. Given that *Bd* imposes strong selective pressure, we might therefore expect directional selection for one or a subset of MHC alleles to predominate in our system. Alternately, selection from other pathogens or different evolutionary forces such as sexual selection may contribute to MHC variation and produce an overall pattern of balancing selection. In our study, we found evidence for directional selection in two populations with high *Bd* mortality (CIC and TV), demographic expansion that has probably resulted from directional selection for allele Q and ST4 in two populations with infection but no *Bd* mortality (HR and SS), and no support for balancing selection acting to equalize frequencies of MHC supertypes in any population (figure 3). Continued population monitoring in future generations may provide direct evidence for the benefits of particular supertypes if the expected changes in *Bd* susceptibility occur. Among our sampled populations, AC has the highest likelihood of evolving future disease resistance, as this population currently harbours allele Q at low frequency, experiences moderate *Bd* mortality, and showed the highest proportion of *Bd* survival in previous laboratory trials [11]. Interestingly, all three population differentiation outliers involved population TV, which was undifferentiated from 0% mortality populations based on MHC supertypes but exceptionally distinct from another high mortality population CIC (figure 4). Further, frogs sampled alive from TV all had susceptibility ST1 and those found dead all had ST2, despite the overall survival disadvantage we found for ST1. MHC dynamics are therefore highly unusual in this population, highlighting that allelic advantages may be unique within populations due to eco-immunological differences in the host or the pathogen under distinct environmental regimes [73]. Excluding these three outlier comparisons involving TV, concordance between MHC- and microsatellite-based differentiation measures demonstrate that demographic processes have played a significant role in shaping MHC evolution. Recent positive selection on MHC codons and strong MHC allelic associations with *Bd* survival may therefore be modest drivers of overall population genetic change compared with forces such as drift and population bottlenecks in a species facing ongoing habitat loss and competition from invasive species [67].

Global declines caused by chytridiomycosis have had catastrophic consequences for amphibian diversity [4] and *Bd* continues to spread to new regions and hosts [74]. Emerging fungal diseases of wildlife are also on the rise, including white-nose syndrome in bats, fungal skin disease in snakes, and honeybee colony collapse disorder [1,2]. Our study highlights the importance of examining fine-scale demographic, epidemiological, and genetic patterns if we are to elucidate the key processes underlying infectious disease dynamics and evolution of resistance in free-living wildlife populations. Identifying immunogenetic correlates of chytridiomycosis outcomes provides a mechanism to explain variable host susceptibility among individuals, populations, and species. Analyses of *Bd* infection dynamics within and across amphibian species, life-history traits, and geographical regions [7–9], may also benefit from the incorporation of data on host genetic variation [17]. Finally, identifying immunogenetic hallmarks of *Bd* resistance in natural populations is a critical step towards species recovery, as the global spread and persistence of *Bd* means that wild populations must ultimately evolve disease resistance to achieve long-term species survival.

## Supplementary Material

Electronic supplementary material

## Data accessibility

DNA sequences: GenBank accession numbers KU877031-KU877108. Microsatellite genotyping data for *Lithobates yavapaiensis* populations available from Dryad Digital Repository http://dx.doi.org/10.5061/dryad.3qk53 and also from [17].
