# Supplementary material for: Adaptive tolerance to a pathogenic fungus drives major histocompatibility complex evolution in natural amphibian populations
Source: Proc Biol Sci. 2016 Mar 30;283(1827):20153115. doi: 10.1098/rspb.2015.3115 (PMC4822461; doi:10.1098/rspb.2015.3115)
Supplement: Electronic supplementary material [file rspb20153115supp1.docx]

**ESM Table S1.** Class II MHC sequences recovered using cloning and Sanger sequencing compared to 454 amplicon sequencing.

|  |  | **454 sequencing** | | **Cloning & Sanger sequencing** | |
| --- | --- | --- | --- | --- | --- |
| ***Individual ID*** | ***Population*** | ***AlleleA*** | ***AlleleB*** | ***AlleleA*** | ***AlleleB*** |
| AES207 | CIC | **Allele_HH** | **Allele_HH** | Allele_VV | Allele_WW |
| AES204 | HR | **AlleleQ** | **AlleleQ** | Allele_SS | Allele_TT |
| AES345 | HR | AlleleQ | **Var_18** | AlleleQ | Allele_07 |
| AES401 | SM | AlleleN | AlleleN | AlleleN | AlleleN |
| AES218 | TV | AlleleN | AlleleN | AlleleN | AlleleN |
| AES529 | TV | AlleleN | AlleleN | AlleleN | AlleleN |
| AES530 | TV | AlleleN | AlleleN | AlleleN | AlleleN |
| AES531 | TV | AlleleN | AlleleN | AlleleN | AlleleN |
| AES535 | TV | AlleleN | AlleleN | AlleleN | AlleleN |
| AES536 | TV | AlleleN | AlleleN | AlleleN | AlleleN |
| AES538 | TV | AlleleN | AlleleN | AlleleN | AlleleN |
| AES539 | TV | AlleleN | AlleleN | AlleleN | AlleleN |
| AES541 | TV | AlleleN | AlleleN | AlleleN | AlleleN |
| AES542 | TV | AlleleN | AlleleN | AlleleN | AlleleN |
| AES048 | TV | AlleleN | AlleleN | AlleleN | AlleleN |
| AES217 | TV | AlleleN | AlleleN | AlleleN | AlleleN |
| AES581 | HR | AlleleQ | AlleleQ | AlleleQ | AlleleQ |
| AES582 | HR | AlleleQ | AlleleQ | AlleleQ | AlleleQ |
| AES583 | HR | AlleleQ | AlleleQ | AlleleQ | AlleleQ |
| AES585 | HR | AlleleQ | AlleleQ | AlleleQ | AlleleQ |
| AES203 | HR | AlleleQ | AlleleQ | AlleleQ | AlleleQ |
| AES592 | MR | Allele_SS | Allele_SS | Allele_SS | Allele_SS |
| AES597 | CIC | Allele_HH | Allele_HH | Allele_HH | Allele_HH |
| AES599 | CIC | Allele_HH | Allele_HH | Allele_HH | Allele_HH |
| AES600 | CIC | Allele_HH | Allele_HH | Allele_HH | Allele_HH |
| AES601 | CIC | Allele_HH | Allele_HH | Allele_HH | Allele_HH |
| AES604 | CIC | Allele_HH | Allele_HH | Allele_HH | Allele_HH |
| AES598 | CIC | Allele_HH | Allele_31 | Allele_HH | Allele_31 |
| AES603 | CIC | Allele_HH | Allele_32 | Allele_HH | Allele_32 |

**ESM Figure S2.** Output from Discriminant Analysis of Principal Components (DAPC) based on physiochemical properties of peptide-interacting codons showing the four recovered MHC supertypes.

**ESM Table S3.** MHC risk factors associated with *Bd* mortality.

| **MHC Risk Factor** | **Relative Risk*** | ***P*-value^†^** | **95% CI** |
| --- | --- | --- | --- |
| Heterozygote | 1.01 | 0.99 | 0.37 – 2.04 |
| ST1 | ***2.80*** | ***0.004*** | ***1.28 – 7.01*** |
| ST2 | 0.60 | 0.12 | 0.29 – 1.15 |
| ST3  ST4 | 1.36  0 | 0.59  0.06 | 0.24 – 2.59  0 – 1.26 |
| Allele A | ***3.20*** | ***<0.0001*** | ***2.10 – 4.79*** |
| Allele N | 0.51 | 0.091 | 0.20 – 1.14 |
| Allele Q | **0.23** | **0.008** | **0.04 – 0.86** |

*Significant values (after Bonferroni correction) are shown in bold

**^†^**Two-tailed Fisher exact test *P*-values

**ESM Table S4.** Population genetic signatures of selection and variability.

| **Population** | **Genetic region** | **E-W test**  ***P*-value** | **Pi** | **Theta (S)** |
| --- | --- | --- | --- | --- |
| *Populations with mortality* | | |  |  |
| CIC^†^ | MHC exon | **1.00** | 2.97 | 7.89 |
| (*N*=24) | MHC intron | -- | 1.70 | 3.15 |
|  | Microsatellites | 0.73 |  |  |
| MR^†^ | MHC exon | 0.65 | 18.48 | 9.76 |
| (*N*=15) | MHC intron | -- | 2.206 | 2.39 |
|  | Microsatellites | 0.92 |  |  |
| TV | MHC exon | **0.99** | 2.72 | 5.96 |
| (*N*=15) | MHC intron | -- | 1.52 | 3.48 |
|  | Microsatellites | 0.87 |  |  |
| WC^†^ | MHC exon | 0.84 | 10.43 | 7.84 |
| (*N*=8) | MHC intron | -- | 3.79 | 3.14 |
|  | Microsatellites | 0.73 |  |  |
| AC***^‡^*** | MHC exon | **0.99** | 20.85 | 27.51 |
| (*N*=20) | MHC intron | -- | 2.63 | 3.33 |
|  | Microsatellites | **0.99** |  |  |
| *Populations without mortality* | | |  |  |
| HR | MHC exon | **1.00** | 3.46 | 8.81 |
| (*N*=19) | MHC intron | -- | 2.10 | 4.05 |
|  | Microsatellites | **0.99** |  |  |
| SM***^‡^*** | MHC exon | **1.00** | 8.69 | 13.11 |
| (*N*=14) | MHC intron | -- | 2.55 | 2.57 |
|  | Microsatellites | **0.99** |  |  |
| SS | MHC exon | 0.76 | 17.34 | 14.54 |
| (*N*=11) | MHC intron | -- | 5.27 | 4.66 |
|  | Microsatellites | 0.92 |  |  |

* Bold values indicate a significant signature of directional selection (*P* > 0.95 for E-W tests and *P* < 0.05 for Tajima’s *D* and Fu’s *F_S_* tests), and italicized values indicate discordance among test results for different genetic markers.

^†^ Experimentally *Bd*-infected population with 100% mortality in the lab

***^‡^*** Experimentally *Bd*-infected population with surviving individuals in the lab
